# Long-term efficacy, clinical outcomes, and safety of vimseltinib in patients with tenosynovial giant cell tumor: results from the MOTION phase 3 trial

**DOI:** 10.1093/oncolo/oyag421

**Published:** 2026-10-19

**Authors:** Vivek Bhadri, Hans Gelderblom, Silvia Stacchiotti, Sebastian Bauer, Andrew J Wagner, Michiel van de Sande, Nicholas M Bernthal, Antonio López Pousa, Albiruni Abdul Razak, Antoine Italiano, Mahbubl Ahmed, Axel Le Cesne, Gabriel Tinoco, Kjetil Boye, Javier Martín-Broto, Emanuela Palmerini, Salvatore Tafuto, Sarah Pratap, Benjamin C Powers, Peter Reichardt, Antonio Casado Herráez, Piotr Rutkowski, Christopher Tait, Giulia Agnello, Brooke Harrow, Amanda Saunders, Maitreyi G Sharma, Matthew L Sherman, Jean-Yves Blay, William D Tap

**Affiliations:** Department of Medical Oncology, Chris O’Brien Lifehouse, Camperdown, NSW 2050, Australia; Department of Medical Oncology, Leiden University Medical Center, Leiden, ZA 2333, The Netherlands; Department of Medical Oncology, Fondazione IRCCS Istituto Nazionale dei Tumori, Milano, 20133, Italy; Department of Medical Oncology and Sarcoma Center/West German Cancer Center, University Hospital Essen, University Duisburg-Essen, Essen, 45147, Germany; German Cancer Consortium (DKTK), Partner Site University Hospital Essen, Essen, 45147, Germany; Department of Medical Oncology, Dana-Farber Cancer Institute, Boston, MA 02215, United States; Department of Medical Oncology, Leiden University Medical Center, Leiden, ZA 2333, The Netherlands; Department of Orthopaedic Surgery, University of California Los Angeles, Los Angeles, CA 90095, United States; Department of Medical Oncology, Hospital de la Santa Creu i Sant Pau, Barcelona, 08041, Spain; Medical Oncology Sarcoma Program, Princess Margaret Cancer Centre, Toronto, ON M5G 2C1, Canada; Department of Medical Oncology, Institut Bergonié and University of Bordeaux, Bordeaux, 33000, France; Department of Medical Oncology, University College London Hospitals NHS Foundation Trust, London, NW1 2PG, United Kingdom; Department of Cancer Medicine, Gustave Roussy, Villejuif, 94800, France; Division of Medical Oncology, The Ohio State University, Columbus, OH 43210, United States; Department of Oncology, Oslo University Hospital, Oslo, 0424, Norway; Department of Medical Oncology, Instituto de Investigactión Sanitaria Fundación Jiménez Díaz (IIS, FJD, UAM), Fundación Jiménez Díaz University Hospital, University Hospital General de Villalba, Madrid, 28400, Spain; Department of Chemotherapy for Rare Musculoskeletal Tumors, IRCCS Istituto Ortopedico Rizzoli, Bologna, 40136, Italy; Sarcomas and Rare Tumors Unit, Istituto Nazionale Tumori IRCCS Fondazione “G.Pascale”, Naples, 80131, Italy; Oxford Cancer and Haematology Centre, Churchill Hospital, Oxford University Hospitals NHS Foundation Trust, Oxford, OX3 9DU, United Kingdom; Medical Oncology Division, University of Kansas Cancer Center, Overland Park, KS 62210, United States; Department of Interdisciplinary Oncology, HELIOS Klinikum Berlin-Buch, Berlin, 13125, Germany; Department of Medical Oncology, Hospital Universitario San Carlos, Madrid, 28040, Spain; Department of Soft Tissue/Bone Sarcoma and Melanoma, Maria Sklodowska-Curie National Research Institute of Oncology, Warszawa, 02-781, Poland; Deciphera Pharmaceuticals, LLC, Waltham, MA 02451, United States; Deciphera Pharmaceuticals, LLC, Waltham, MA 02451, United States; Deciphera Pharmaceuticals, LLC, Waltham, MA 02451, United States; Deciphera Pharmaceuticals, LLC, Waltham, MA 02451, United States; Deciphera Pharmaceuticals, LLC, Waltham, MA 02451, United States; Deciphera Pharmaceuticals, LLC, Waltham, MA 02451, United States; Department of Medical Oncology, Centre Léon Bérard, Lyon, 69008, France; Sarcoma Medical Oncology Service, Memorial Sloan Kettering Cancer Center and Weill Cornell Medical College, New York, NY 10065, United States

**Keywords:** giant cell tumor of tendon sheath, tyrosine kinase inhibitors, clinical trial, treatment outcomes

## Abstract

**Background:**

Tenosynovial giant cell tumor (TGCT) is a locally aggressive neoplasm caused by colony-stimulating factor 1 (*CSF1*) gene dysregulation. Vimseltinib is an oral, switch-control tyrosine kinase inhibitor of the CSF1 receptor. In part 1 of the MOTION phase 3 trial, vimseltinib demonstrated statistically significant and clinically meaningful improvements in antitumor activity and clinical outcome assessments (COAs). Here, we report long-term results from MOTION in patients with >2 years of follow-up on study.

**Methods:**

MOTION is a randomized, double-blind, placebo-controlled, phase 3 trial with double-blind (part 1; to week 25), open-label (part 2; weeks 25-49), and extension periods (NCT05059262). Eligible adult patients with histologically confirmed TGCT not amenable to surgery received vimseltinib 30 mg twice weekly. Efficacy was assessed by objective response rate (ORR) on study evaluated by independent radiological review per Response Evaluation Criteria in Solid Tumors version 1.1 (RECIST v1.1) and Tumor Volume Score. COAs and safety were assessed.

**Results:**

In total, 118 patients received vimseltinib during MOTION (randomized to vimseltinib, *n* = 83; crossover, *n* = 35), with 51% (60/118) remaining on treatment. ORR on study per RECIST v1.1 was 48% (40/83) for patients randomized to vimseltinib and 54% (19/35) for crossover. Patients in both groups experienced long-term improvements in COAs. The safety profile was consistent with prior reports, with no evidence of cholestatic hepatotoxicity or drug-induced liver injury.

**Conclusion:**

Vimseltinib continued to provide robust and durable antitumor activity and demonstrated long-term improvements in COAs, with manageable safety for the treatment of patients with TGCT not amenable to surgery.

Implications for practice This article reports long-term efficacy and safety from the MOTION phase 3 trial of vimseltinib in patients with tenosynovial giant cell tumor for whom surgery was unlikely to provide benefit. The results demonstrated that vimseltinib provided long-term tumor shrinkage; improved range of motion and patient-reported physical function, stiffness, pain, and quality of life; and had a manageable safety profile. This study showed that treatment with vimseltinib is effective, and the article helps inform patients, caregivers, and physicians of the benefit received from continued treatment with vimseltinib.

## Introduction

Tenosynovial giant cell tumor (TGCT), historically referred to as pigmented villonodular synovitis or giant cell tumor of the tendon sheath, is a locally aggressive neoplasm that arises within the synovium, bursae, and tendon sheath.^1^ Dysregulated expression of the colony-stimulating factor 1 (*CSF1*) gene leads to increased CSF1 protein levels, which drive disease pathogenesis.^1–3^ The resulting overproduction of CSF1 attracts and promotes the proliferation of CSF1 receptor (CSF1R)-dependent inflammatory macrophages, which contribute to tumor expansion and progression.^4^^,^^5^

While surgery remains the standard of care for TGCT management, some patients have disease that cannot be effectively managed with surgery because additional procedures are unlikely to be beneficial or would result in considerable morbidity and functional limitations.^1^^,^^6^ The diffuse subtype of TGCT is more often assessed as being not amenable to surgery compared with the localized subtype.^7^^,^^8^ TGCT can be associated with a substantial reduction in quality of life, with some patients experiencing joint damage that requires total joint replacement or, in extreme cases, amputation.^1^^,^^9^ Up to 72% of patients with diffuse TGCT experience disease recurrence, and those who have a recurrence are at significantly higher risk of experiencing subsequent recurrences.^10–12^ Furthermore, the mean age at diagnosis is 35-50 years, suggesting that patients living with TGCT may be impacted for substantial periods of time.^1^^,^^12^

Targeting CSF1/CSF1R signaling with systemic therapies is an effective treatment strategy for patients with symptomatic TGCT not amenable to surgery. Currently, the kinase inhibitors pexidartinib, pimicotinib, and vimseltinib are approved for use in patients with TGCT in various regions. From 2019 to 2025, pexidartinib was the only systemic treatment approved in the United States, South Korea, and Taiwan for the treatment of TGCT associated with severe morbidity or functional limitations not amenable to improvement with surgery.^13–15^ In December 2025, pimicotinib was approved by the China National Medical Products Administration for the treatment of adult patients with symptomatic TGCT for which surgical resection will potentially cause functional limitation or relatively severe morbidity.^16^ Based on the results of the MOTION phase 3 trial, vimseltinib, an oral, switch-control CSF1R inhibitor, was first approved in February 2025 by the US Food and Drug Administration for the treatment of adult patients with symptomatic TGCT for which surgical resection will potentially cause worsening functional limitation or severe morbidity.^2^^,^^17–19^ In September 2025, the European Medicines Agency also approved vimseltinib for use in adult patients with symptomatic TGCT associated with clinically relevant physical function deterioration and in whom surgical options have been exhausted or would induce unacceptable morbidity or disability.^20^ In the primary analysis of MOTION, vimseltinib provided statistically significant and clinically meaningful improvements vs placebo in tumor response as well as in active range of motion (ROM) and patient-reported physical function, stiffness, health status, and pain.^19^

Here, we report the final analysis from the MOTION phase 3 trial, which includes efficacy, clinical outcome assessments (COAs), and safety, with all patients having at least 2 years of follow-up on study.

## Methods

### Trial design and patients

MOTION is a phase 3, global, double-blind, randomized, placebo-controlled trial. Part 1 was the double-blind portion (to week 25), part 2 was the open-label portion (weeks 25-49), and after week 49 patients could continue on study in an extension period (NCT05059262).^19^ The methods for this study were described previously in the primary analysis (part 1; data cutoff: August 22, 2023).^19^ Briefly, adult patients with symptomatic TGCT and a histologically confirmed diagnosis for whom surgical resection would potentially cause worsening functional limitation or severe morbidity, as determined by surgical consultation or multidisciplinary tumor board, were enrolled. Eligible patients had at least 1 measurable lesion ≥2 cm using Response Evaluation Criteria in Solid Tumors version 1.1 (RECIST v1.1) as assessed by magnetic resonance imaging by a central radiologist. Exclusion criteria included receiving prior specific CSF1/CSF1R inhibitor therapy and active liver or biliary disease.

This study was conducted in accordance with the Declaration of Helsinki and Good Clinical Practice guidelines. Applicable local and regulatory requirements were followed. The protocol, protocol amendments, and informed consent documents were approved by an independent institutional review board or ethics committee at each site and by appropriate regulatory authorities. All patients provided written informed consent.

### Randomization and treatment

In part 1, patients were randomized 2:1 using interactive response technology to receive vimseltinib or matching placebo in 28-day cycles for 24 weeks. After 24 weeks, patients could continue on or cross over to receive vimseltinib in part 2 (weeks 25-49), after which patients could continue to receive vimseltinib in the extension period. Vimseltinib capsules were administered orally with an initial dose of 30 mg twice weekly on days 1 and 5 (±1 day) each week at approximately the same time of day on an empty stomach, with at least 24 hours between doses. The dose of vimseltinib could be modified due to adverse events (AEs) at the discretion of the investigator. Two dose level reductions were allowed (to 20 and 14 mg), after which study drug was discontinued if more than 2 dose level reductions were required. In parts 1 and 2, patients attended study visits on days 1 and 15 of cycle 1 and on day 1 (±7 days) for all subsequent cycles. Starting in the extension period (after week 49), the schedule of assessments changed, with patients having study visits on day 1 (±7 days) of every 3 cycles (12 weeks).

### Outcomes

The primary and key secondary endpoints at week 25 were previously reported in the primary analysis.^19^ Results from this updated analysis are reported with at least 2 years of follow-up on study in all patients. For patients who crossed over to vimseltinib, the time on treatment may be less than 2 years due to the time on study receiving placebo. Efficacy was assessed by objective response rate (ORR) on study (complete response + partial response), which is based on best overall response by independent radiological review (IRR) per RECIST v1.1 and per Tumor Volume Score (TVS),^21^^,^^22^ as well as duration of response (DOR). DOR was defined as the time from first complete or partial response to disease progression or death, whichever came first. COAs reported here are change from baseline in active ROM, Patient-Reported Outcomes Measurement Information System Physical Function (PROMIS-PF; TGCT-specific), worst stiffness numeric rating scale (NRS), EuroQol Visual Analogue Scale (EQ-VAS), and Brief Pain Inventory (BPI) worst pain. Worst stiffness NRS and BPI worst pain data are only included up to week 49 due to the change in frequency of study visits. In the first 49 weeks, patients were asked the BPI worst pain question every day and were asked to rate their worst stiffness every other day for the first 3 weeks of every cycle. After week 49, patients were only asked about worst pain and worst stiffness once a month; therefore, the results are not comparable to the averaged results in the first 49 weeks. Minimum clinically important difference thresholds for all patient-reported outcomes were derived from a mixed method including anchor- and distribution-based methods (≥10% for active ROM, ≥3 on PROMIS-PF, ≤ −2 on worst stiffness NRS, and ≥7 on EQ-VAS).^19^^,^^23^ Safety was assessed using the National Cancer Institute Common Terminology Criteria for AEs, version 5.0.

### Statistics

This analysis includes at least 2 years of follow-up on study for all patients who received vimseltinib (data cutoff: February 22, 2025). Baseline for COAs (active ROM, PROMIS-PF, worst stiffness NRS, EQ-VAS, and BPI worst pain) was defined at study entry, and for all other assessments, baseline was the last assessment prior to the first dose of vimseltinib. The intent-to-treat population included all patients who were randomized to a treatment for baseline and COA analyses. The safety population included all patients who received at least 1 dose of vimseltinib. Continuous variables were summarized using descriptive statistics, and categorical variables were summarized using frequencies and proportions. DOR and time to treatment discontinuation were summarized by the Kaplan–Meier method.

## Results

### Patients

In total, 118 patients received vimseltinib in the MOTION study (Figure 1). In part 1, 83 patients were randomized to vimseltinib and 73 of these patients continued to receive vimseltinib in part 2. Of the 40 patients randomized to placebo in part 1, 35 received vimseltinib in the open-label part 2. The median treatment duration (range) was 23.6 months (2-36) and 19.1 months (1-30) for patients randomized to vimseltinib and for those who crossed over from placebo to vimseltinib, respectively. At data cutoff, 51% (60/118) of patients remained on treatment. Reasons for treatment discontinuation were withdrawal by patient (*n* = 29), AE (*n* = 14), physician decision (*n* = 3), noncompliance with study drug (*n* = 2), progressive disease by IRR (*n* = 2), unrelated death (*n* = 1 [reported reason due to “fall”]), and other (*n* = 7).

**Figure 1.**
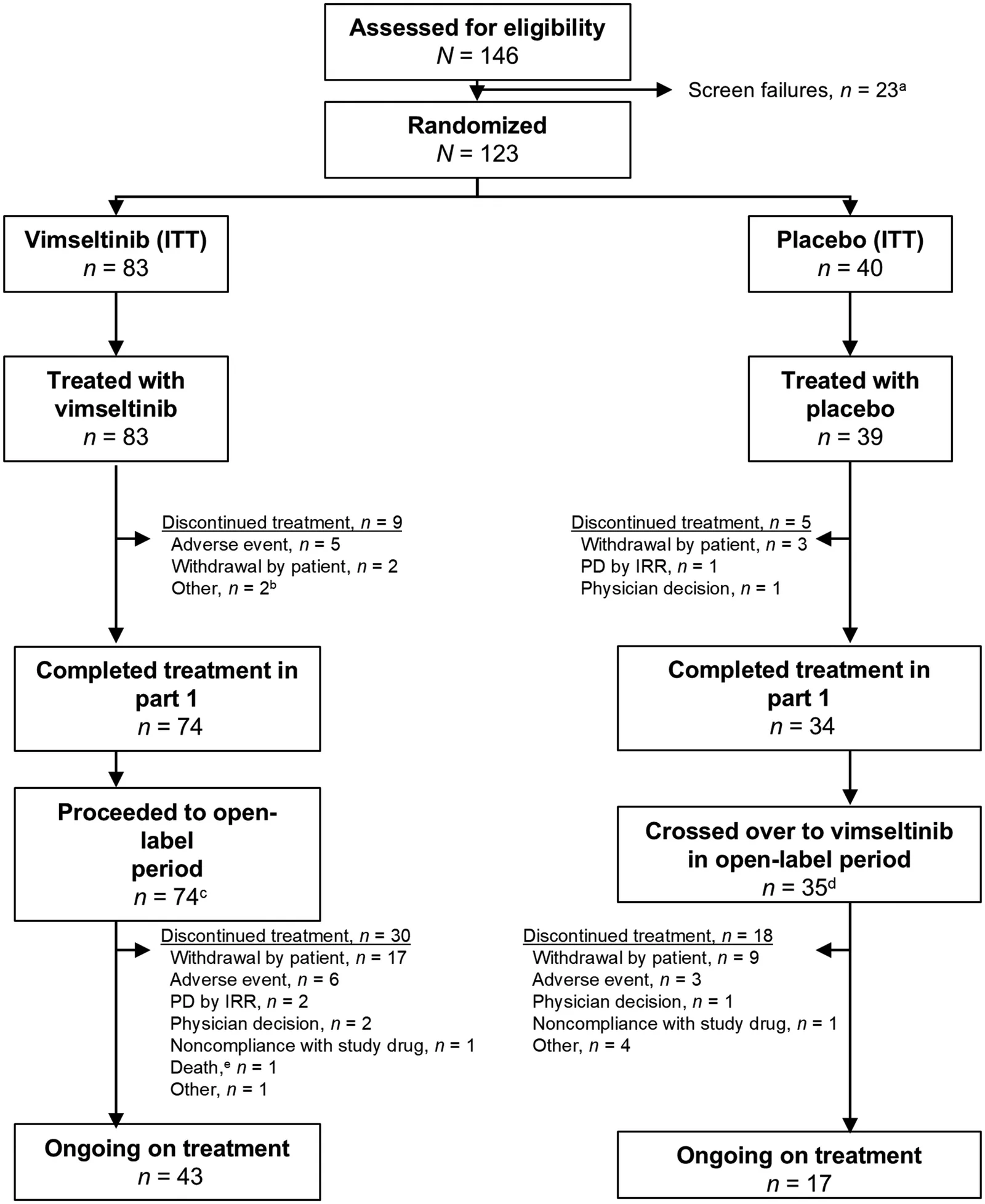
CONSORT flow diagram. Data from the ITT population. Abbreviations: IRR, independent radiological review; ITT, intent-to-treat; PD, progressive disease. ^a^Reasons for exclusion at screening were not captured in the database. ^b^Both patients in this category specified lack of clinical benefit as the reason for withdrawal. ^c^One patient did not receive treatment in part 2. ^d^One patient discontinued treatment early in part 1 and crossed over to receive vimseltinib in the open-label period (part 2). ^e^Unrelated death; reported reason due to “fall.”

Baseline characteristics and demographics by randomized assignment were previously reported and are shown in Table 1.^19^ The median age (interquartile range) was 45 years (33-53) for patients randomized to vimseltinib and 43 years (31-53) for those randomized to placebo, and patients most frequently had TGCT of the knee (randomized to vimseltinib, 67%; randomized to placebo, 68%). Most patients (randomized to vimseltinib, 77%; randomized to placebo, 68%) had at least 1 prior surgery or procedure for TGCT.

**Table 1.** Patient demographics and baseline clinical characteristics.

	Randomized to vimseltinib	Randomized to placebo
	*n* = 83	*n* = 40
**Age, median (IQR), years**	45 (33-53)	43 (31-53)
**Sex, *n* (%)**		
** Female**	46 (55)	27 (68)
** Male**	37 (45)	13 (33)
**Race, *n* (%)**		
** White**	59 (71)	21 (53)
** Asian**	1 (1)	4 (10)
** Black or African American**	4 (5)	0
** Other^a^**	19 (23)	15 (38)
**Affected joint, *n* (%)**		
** Knee**	56 (67)	27 (68)
** Ankle**	9 (11)	6 (15)
** Hip**	11 (13)	1 (3)
** Other^b^**	7 (8)	6 (15)
**Prior TGCT surgery or procedure,^c^ *n* (%)**	64 (77)	27 (68)
**Prior TGCT systemic therapy, *n* (%)**	19 (23)	9 (23)^d^
** Imatinib**	16 (19)	7 (18)
** Nilotinib**	2 (2)	4 (10)
** Other^e^**	1 (1)	0

Data from the ITT population. Percentages may not total to 100 due to rounding.

Abbreviations: ITT, intent-to-treat; TGCT, tenosynovial giant cell tumor.

a Includes not reported and unknown.

b Includes foot, wrist, hand, shoulder, elbow, and temporomandibular joint.

c All patients had histologically confirmed TGCT per diagnostic biopsy or existing pathology report; diagnostic biopsies were not recorded as a previous surgery or procedure.

d Two patients in the placebo group received both imatinib and nilotinib.

e Includes an investigational agent (BP 27 672).

### Efficacy

With a median follow-up while receiving vimseltinib of 27 months for patients randomized to vimseltinib and 20 months for those who crossed over from placebo to vimseltinib, ORR on study per RECIST v1.1 was 48% (40/83) for patients randomized to vimseltinib and 54% (19/35) for those who crossed over (Table 2). Complete responses occurred in 23% (19/83) of patients randomized to vimseltinib and 11% (4/35) of patients who crossed over to vimseltinib. The median DOR was still not reached for both groups (range: vimseltinib, 0.03+ to 30.9+ months; crossover, 0.03+ to 25.4+ months); the + symbols indicate that responses were still ongoing at the time of last assessment. For patients with objective responses per RECIST v1.1, 14% (8/59) had responses occur after 1 year of treatment or longer and 14% (8/59) had responses continue after treatment discontinuation (Figure 2).

**Figure 2.**
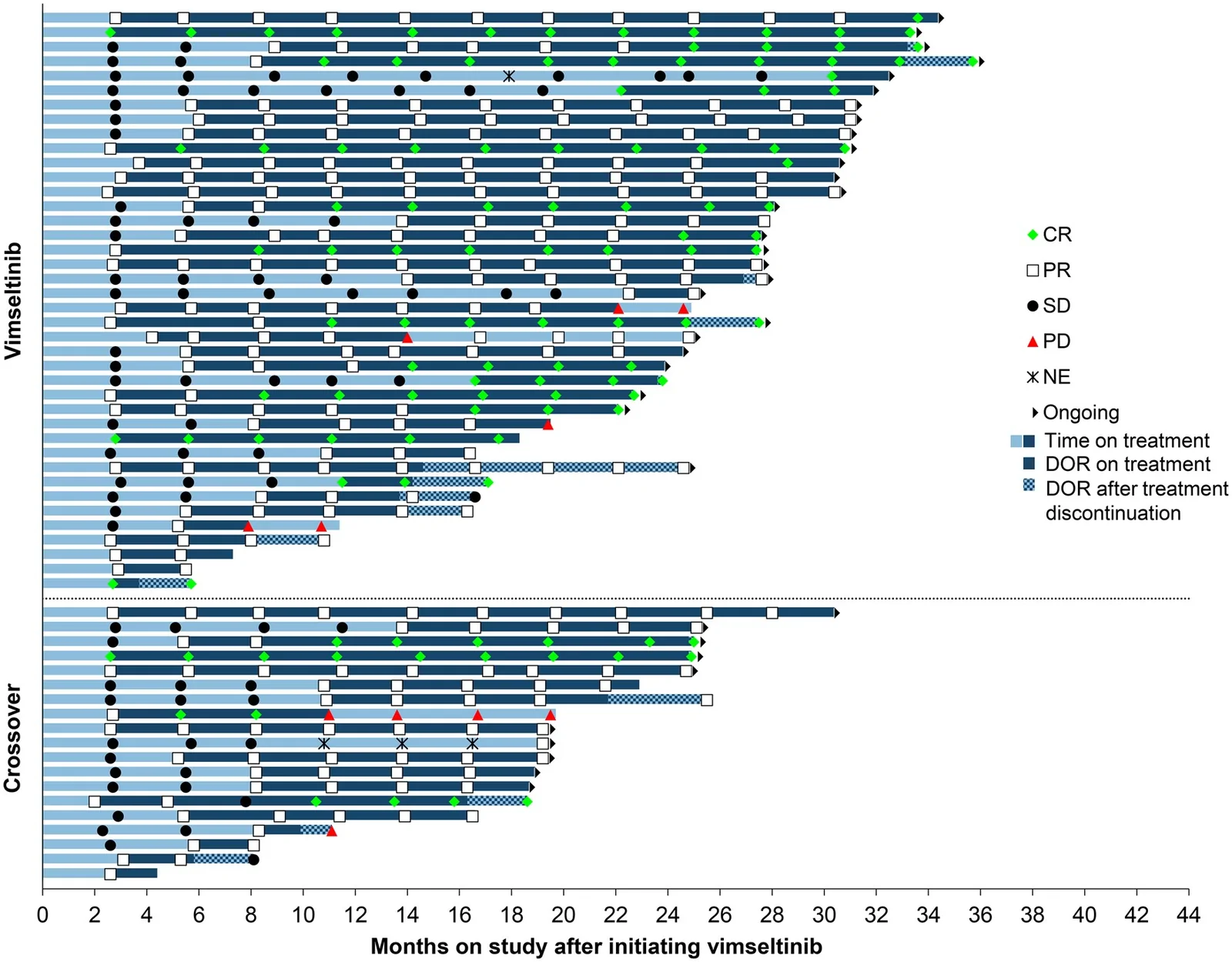
Time on treatment and DOR in patients receiving vimseltinib with objective responses per RECIST v1.1. Data cutoff: February 22, 2025. Data from the ITT population. Dark blue and patterned shading represents the DOR. Abbreviations: CR, complete response; DOR, duration of response; ITT, intent-to-treat; NE, not evaluable; PD, progressive disease; PR, partial response; RECIST v1.1, Response Evaluation Criteria in Solid Tumors version 1.1; SD, stable disease.

**Table 2.** Antitumor response and clinical outcome assessments.

	Randomized to vimseltinib	Crossed over to vimseltinib
	*n* = 83	*n* = 35
**RECIST v1.1**		
** Best overall response, *n* (%)**		
** Complete response**	19 (23)	4 (11)
** Partial response**	21 (25)	15 (43)
** Stable disease**	43 (52)	14 (40)
** Not evaluable**	0	2 (6)
** ORR, *n* (%) [95% CI]**	40 (48) [37 to 59]	19 (54) [37 to 71]
** DOR, median (range), months**	NR (0.03+ to 30.9+)	NR (0.03+ to 25.4+)
**TVS**		
** Best overall response, *n* (%)**		
** Complete response**	20 (24)	4 (11)
** Partial response**	47 (57)	21 (60)
** Stable disease**	16 (19)	8 (23)
** Not evaluable**	0	2 (6)
** ORR, *n* (%) [95% CI]**	67 (81) [71 to 89]	25 (71) [54 to 85]
** DOR, median (range), months**	NR (2.4+ to 33.1+)	NR (1.9+ to 25.4+)
** Active ROM, mean CFB (SE), percentage points**		
** At week 25 (*n* = 73 and 33)**	19.3 (3.7)	4.2 (4.5)
** At week 49 (*n* = 54 and 26)**	14.9 (5.0)	17.5 (8.7)
** At week 97 (*n* = 41 and 22)**	22.1 (4.0)	60.1 (36.6)
** PROMIS-PF, mean CFB (SE)**		
** At week 25 (*n* = 63 and 30)**	4.6 (0.8)	1.1 (0.8)
** At week 49 (*n* = 44 and 18)**	6.5 (1.2)	4.5 (1.4)
** At week 93 (*n* = 18 and 8)**	6.8 (1.7)	6.6 (2.4)
** EQ-VAS, mean CFB (SE)**		
** At week 25 (*n* = 64 and 30)**	12.0 (2.5)	4.0 (3.2)
** At week 49 (*n* = 44 and 18)**	11.0 (3.5)	9.1 (5.6)
** At week 93 (*n* = 18 and 8)**	11.7 (5.9)	0.5 (2.9)
** Worst stiffness NRS, mean CFB (SE)**		
** At week 25 (*n* = 63 and 27)**	−2.2 (0.3)	−0.6 (0.3)
** At week 49 (*n* = 38 and 17)**	−2.7 (0.4)	−1.8 (0.3)
** BPI worst pain, mean CFB (SE)**		
** At week 25 (*n* = 68 and 31)**	−2.2 (0.3)	−1.0 (0.3)
** At week 49 (*n* = 44 and 23)**	−2.8 (0.4)	−2.0 (0.5)

Data cutoff: February 22, 2025. Data from the ITT population. Efficacy was assessed by independent radiological review. + denotes response was ongoing at the last assessment. Baseline for COAs for all patients (including those who crossed over from placebo to vimseltinib) was the study baseline.

Abbreviations: BPI, Brief Pain Inventory; CFB, change from baseline; COA, clinical outcome assessment; DOR, duration of response; EQ-VAS, EuroQol Visual Analogue Scale; ITT, intent-to-treat; NR, not reached; NRS, numeric rating scale; ORR, objective response rate; PROMIS-PF, Patient-Reported Outcomes Measurement Information System Physical Function; RECIST v1.1, Response Evaluation Criteria in Solid Tumors version 1.1; ROM, range of motion; TVS, Tumor Volume Score.

ORR on study per TVS was 81% (67/83) for patients randomized to vimseltinib and 71% (25/35) for those who crossed over from placebo to vimseltinib. Complete responses occurred in 24% (20/83) of patients randomized to vimseltinib and 11% (4/35) of patients who crossed over to vimseltinib. The median DOR per TVS was also not reached for both groups (range: vimseltinib, 2.4+ to 33.1+ months; crossover, 1.9+ to 25.4+ months), as responses were also ongoing at the time of last assessment.

### Clinical outcome assessments

Vimseltinib provided meaningful long-term improvements in active ROM and patient-reported physical function, stiffness, health status, and pain (Table 2). The mean change from baseline (standard error [SE]) in active ROM at week 97 was 22.1 percentage points (4.0) for patients randomized to vimseltinib and 60.1 percentage points (36.6) for those who crossed over to vimseltinib. Improvements were also observed in PROMIS-PF, with a mean change from baseline (SE) at week 93 of 6.8 (1.7) for patients randomized to vimseltinib and 6.6 (2.4) for those who crossed over to vimseltinib, as well as in EQ-VAS (patients randomized to vimseltinib, 11.7 [5.9]; patients who crossed over to vimseltinib, 0.5 [2.9]). Mean change from baseline (SE) at week 49 in worst stiffness was −2.7 (0.4) for patients randomized to vimseltinib and −1.8 (0.3) for those who crossed over to vimseltinib, and mean change from baseline (SE) in worst pain was −2.8 (0.4) for patients randomized to vimseltinib and −2.0 (0.5) for those who crossed over to vimseltinib. The improvements in COAs with vimseltinib occurred within 9-21 weeks of treatment and were long-lasting both in patients randomized to vimseltinib and in those who crossed over (Figure 3). Additionally, vimseltinib demonstrated improvements in the patient-reported ability to walk for 15 minutes, stand for 1 hour, and walk up and down stairs, as well as how much their TGCT limited moderate work in both patient populations per PROMIS (Figure S1, see online supplementary material for a color version of this figure).

**Figure 3.**
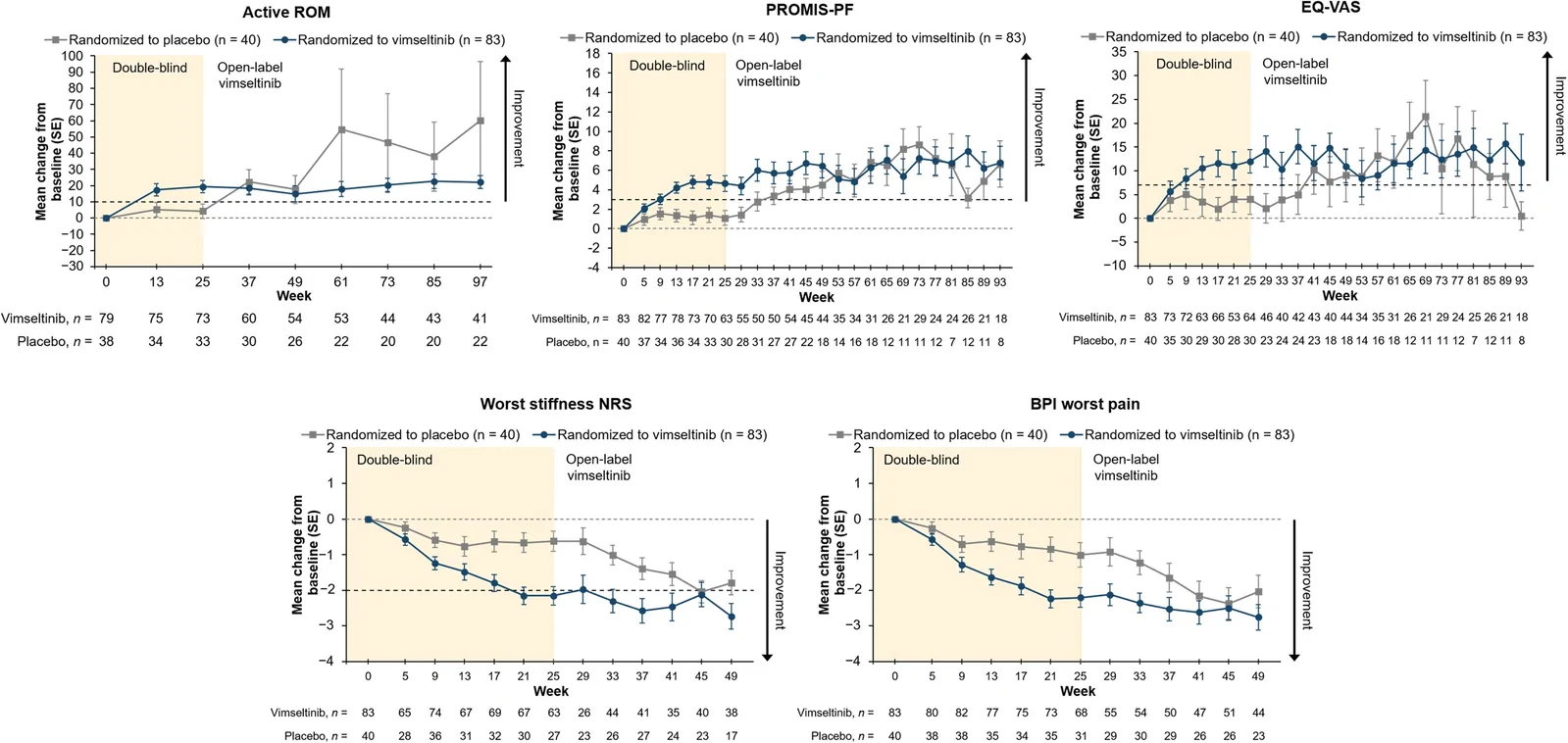
Change in COAs over time. Data cutoff: February 22, 2024 ([D] worst stiffness NRS and [E] BPI worst pain) and February 22, 2025 ([A] active ROM, [B] PROMIS-PF, and [C] EQ-VAS). Data from the ITT population. *n* values reflect the number of patients with data at each time point. Worst stiffness NRS and BPI worst pain data are only included up to week 49 due to the change in frequency of study visits during the extension period. Dashed black lines show the minimum clinically important difference thresholds for each COA (active ROM, 10%; PROMIS-PF, 3; EQ-VAS, 7; and worst stiffness NRS, −2).^19^ Abbreviations: BPI, Brief Pain Inventory; COA, clinical outcome assessment; EQ-VAS, EuroQol Visual Analogue Scale; ITT, intent-to-treat; NRS, numeric rating scale; PROMIS-PF, Patient-Reported Outcomes Information Measurement System Physical Function; ROM, range of motion.

### Safety

Approximately half of patients receiving vimseltinib experienced maximum grade 1/2 treatment-emergent AEs (TEAEs; Table 3, Table S1). Grade 3/4 TEAEs in part 2 were similar between patients randomized to vimseltinib and those who crossed over from placebo to vimseltinib, and grade 3/4 TEAEs occurring in more than 5% of patients were increased blood creatine phosphokinase (16%) and hypertension (8%). Creatine phosphokinase elevations were generally reversible with treatment interruptions, and there was no evidence of cardiac toxicity or rhabdomyolysis. Treatment-related serious TEAEs occurred in 3 patients and were polyarthritis, subcutaneous abscess, and syncope. There was no evidence of cholestatic hepatotoxicity or drug-induced liver injury. The onset of the majority of the most severe AEs (worst grade experienced by a patient) occurred within the first 24 months of treatment (Figure 4).

**Figure 4.**
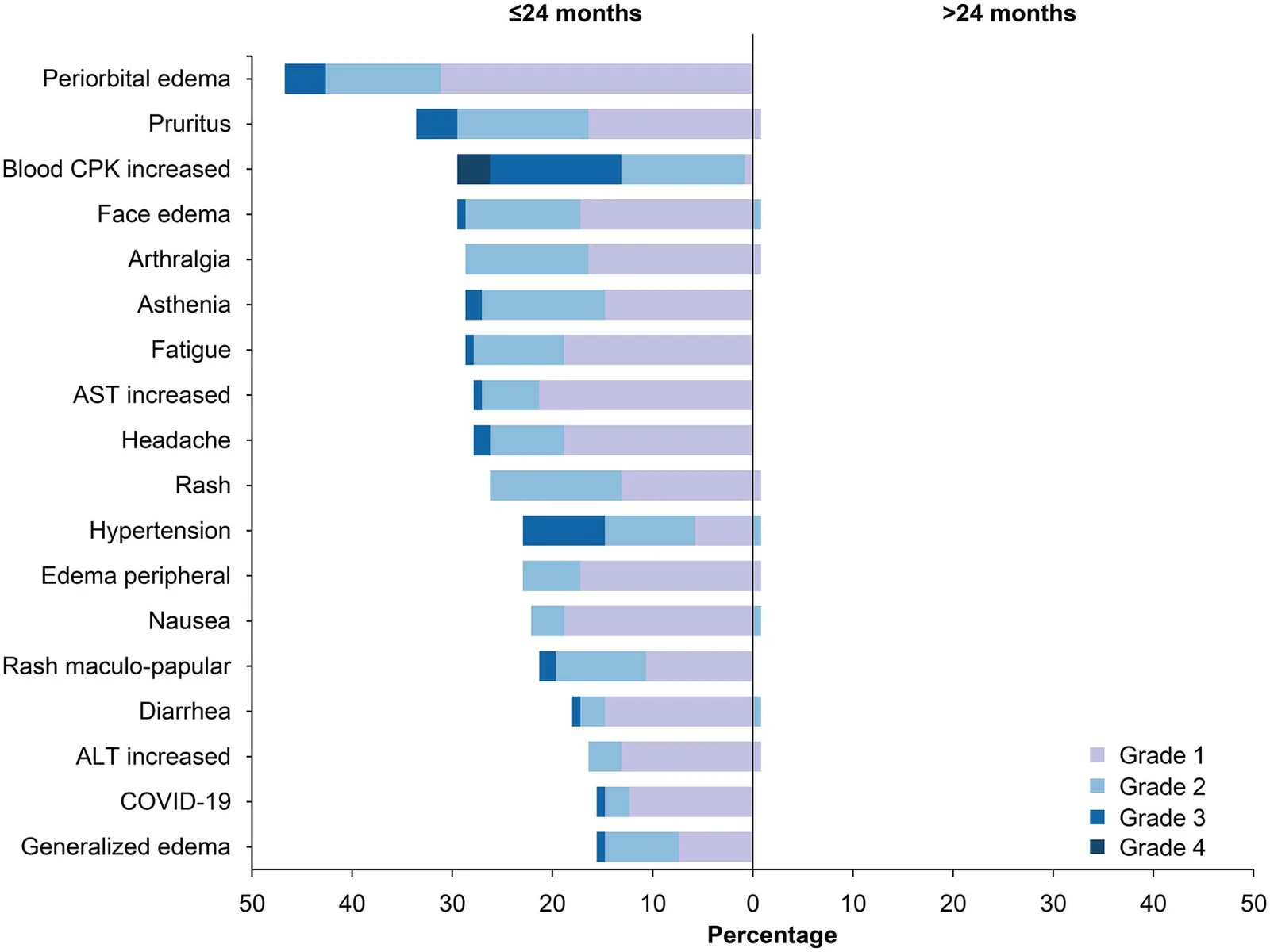
Percentage of TEAEs by maximum grade in ≥10% of patients with onset before and after 24 months on treatment. Data cutoff: February 22, 2025. Data from the safety population. Time denotes the earliest start date of the worst grade for each TEAE; if the highest grade TEAE occurred in the first 24 months of treatment, lower-grade events that occurred after 24 months were not captured in this figure. Abbreviations: ALT, alanine aminotransferase; AST, aspartate aminotransferase; COVID-19, coronavirus disease-2019; CPK, creatine phosphokinase; TEAE, treatment-emergent adverse event.

**Table 3.** TEAEs in ≥15% of patients receiving vimseltinib.

	Randomized to vimseltinib		Crossed over to vimseltinib		Vimseltinib total	
	*n* = 83		*n* = 35		*N* = 118	
Preferred term, *n* (%)	All grades	Grade 3/4	All grades	Grade 3/4	All grades	Grade 3/4
**Periorbital edema^a^**	40 (48)	4 (5)	17 (49)	1 (3)	57 (48)	5 (4)
**Pruritus^a^**	31 (37)	3 (4)	11 (31)	2 (6)	42 (36)	5 (4)
**Face edema^a^**	28 (34)	1 (1)	9 (26)	0	37 (31)	1 (1)
**Arthralgia**	27 (33)	0	9 (26)	0	36 (31)	0
**Blood CPK increased**	26 (31)	12 (14)	10 (29)	7 (20)	36 (31)	19 (16)
**Asthenia^a^**	27 (33)	1 (1)	8 (23)	1 (3)	35 (30)	2 (2)
**Fatigue**	30 (36)	1 (1)	5 (14)	0	35 (30)	1 (1)
**AST increased**	23 (28)	1 (1)	11 (31)	0	34 (29)	1 (1)
**Headache^a^**	25 (30)	1 (1)	9 (26)	1 (3)	34 (29)	2 (2)
**Rash**	27 (33)	0	6 (17)	0	33 (28)	0
**Hypertension**	18 (22)	6 (7)	11 (31)	4 (11)	29 (25)	10 (8)
**Edema peripheral**	21 (25)	0	8 (23)	0	29 (25)	0
**Nausea**	22 (27)	0	6 (17)	0	28 (24)	0
**Rash maculopapular^a^**	20 (24)	2 (2)	6 (17)	0	26 (22)	2 (2)
**Diarrhea**	15 (18)	1 (1)	8 (23)	0	23 (19)	1 (1)
**ALT increased**	13 (16)	0	8 (23)	0	21 (18)	0
**COVID-19**	16 (19)	1 (1)	3 (9)	0	19 (16)	1 (1)
**Generalized edema**	15 (18)	1 (1)	4 (11)	0	19 (16)	1 (1)

Data cutoff: February 22, 2025. Data from the safety population.

Abbreviations: AE, adverse event; ALT, alanine aminotransferase; AST, aspartate aminotransferase; COVID-19, coronavirus disease 2019; CPK, creatine phosphokinase; TEAE, treatment-emergent AE.

a Denotes AEs without grade 4 criteria per Common Terminology Criteria for AEs version 5.0.

Dose interruptions due to TEAEs occurred in 63% (74/118) of patients, and dose reductions due to TEAEs in 58% (68/118); 12% (14/118) of patients discontinued vimseltinib due to TEAEs. The TEAEs that led to treatment discontinuation in more than 1 patient were pruritus (*n* = 3), periorbital edema (*n* = 3), and rash (*n* = 2). Most patients received vimseltinib 30 or 20 mg during the study, having only 1 dose reduction, if any; the median time to treatment discontinuation (range) was 27.8 months (0.8-35.8+; Figure 5).

**Figure 5.**
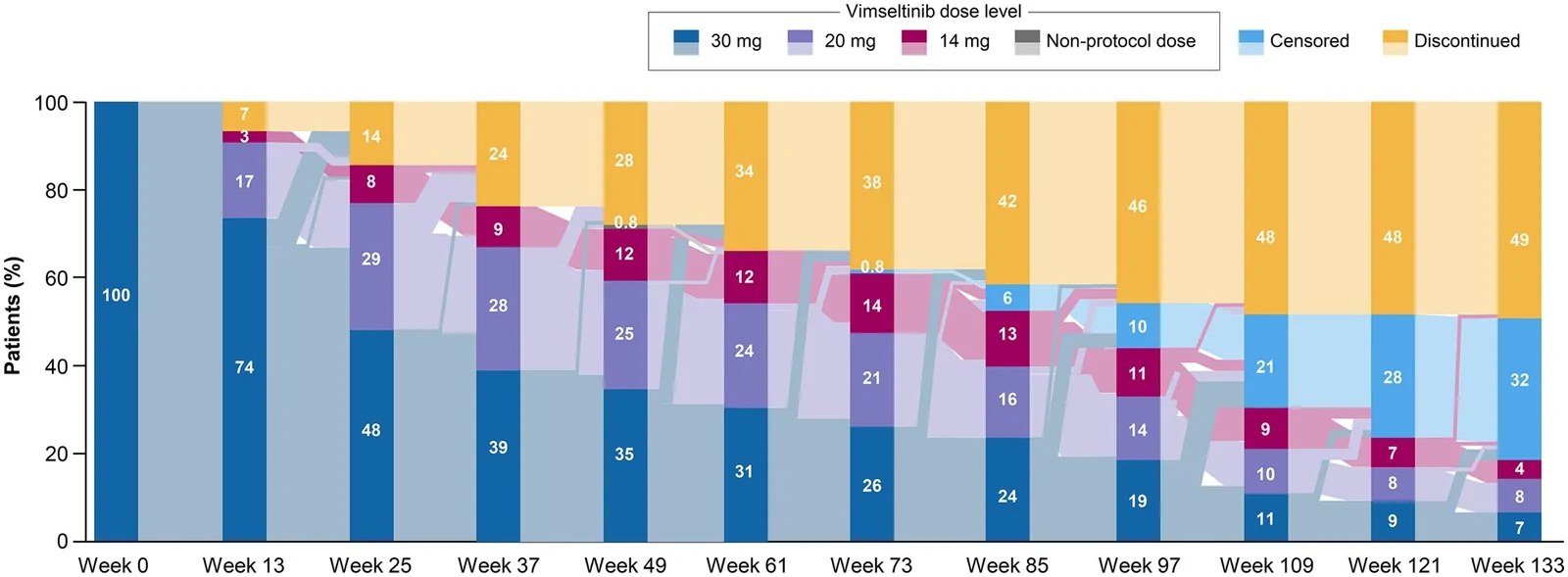
Dose reductions and treatment discontinuations over time. Data cutoff: February 22, 2025. Data from the safety population. A patient was censored if they were ongoing on treatment but had not completed the visit. One patient received a dose of vimseltinib not specified in the protocol at week 49 and was included separately in that week.

## Discussion

This analysis from MOTION included long-term efficacy, COA, and safety results in patients with symptomatic TGCT and disease not amenable to surgery and treated with vimseltinib. Vimseltinib demonstrated robust and long-term antitumor efficacy, long-lasting improvements in clinical outcome assessments, and a manageable safety profile both in patients randomized to vimseltinib and those who crossed over from placebo to vimseltinib, building upon the results from the primary analysis^19^ and supporting the received regulatory approvals.

Efficacy as assessed by ORR on study per RECIST v1.1 with vimseltinib was 48% for patients randomized to vimseltinib and 54% for those who crossed over from placebo to vimseltinib. Results per TVS were robust, with ORR on study of 81% for patients randomized to vimseltinib and 71% for those who crossed over. Similar to the primary analysis from MOTION, volumetric assessment identified a higher proportion of responses compared with RECIST v1.1,^19^ reflecting greater sensitivity to the gradual tumor shrinkage observed with TGCT. Two patients experienced partial responses with vimseltinib after remaining on treatment for over 18 months; both had dose reductions and later met IRR criteria for disease progression due to an increase from nadir. All remaining patients with evaluable tumors had best responses of stable disease per RECIST v1.1 or per TVS. The median DOR was not yet reached at data cutoff, illustrating durable responses with vimseltinib. Furthermore, longer follow-up in MOTION showed deepened responses with vimseltinib, as the ORR increased compared to those at week 25 both per RECIST v1.1 and per TVS.^19^

In this study, vimseltinib showed rapid and durable improvements in range of motion, physical function, health status, stiffness, and pain, both in patients randomized to vimseltinib and in those who crossed over to vimseltinib. For active ROM, PROMIS-PF, and EQ-VAS, the improvements from baseline were sustained for at least 22 months of treatment. Furthermore, the proportion of patients who reported the ability to perform certain tasks “without difficulty” increased over time while receiving vimseltinib, including the ability to walk for 15 minutes, stand for 1 hour, and walk up and down stairs at a normal pace. However, the interpretation of PROMIS-PF and EQ-VAS results is limited, as there were fewer patients with evaluable assessments toward the end of the study. Improvements in stiffness and pain with vimseltinib were observed up to week 49 in both populations, but data past week 49 were not included due to the change in the schedule of assessments. These results demonstrate that vimseltinib provides symptomatic and functional health improvements to a population living with substantial morbidity over extended periods of time.

Consistent with the primary analysis, vimseltinib demonstrated a manageable safety profile with an additional 18 months of follow-up on study in MOTION. Approximately half of the patients experienced maximum grade 1/2 TEAEs, and grade 3/4 TEAEs experienced in more than 5% of patients were increased blood CPK and hypertension. Serum enzyme elevations, including CPK, were consistent with the known mechanism of action of CSF1R inhibition,^24^^,^^25^ and there was no evidence of cholestatic hepatotoxicity, drug-induced liver injury, cardiac toxicity, or rhabdomyolysis. Most patients had only 1 dose reduction during MOTION, and the majority of discontinuations occurred within the first year of treatment. Furthermore, the onset of the majority of grade 3/4 TEAEs occurred within the first 2 years of treatment. With a median follow-up on study of 25 months, 51% of patients remain on treatment, demonstrating the long-term tolerability of treatment with vimseltinib and highlighting the long-term benefit of close monitoring during early treatment. TEAEs led to dose reduction in 58% of patients, but only 12% reported TEAEs as their reason for treatment discontinuation, demonstrating that dose adjustments are often successfully used to clinically manage AEs during treatment with vimseltinib. The TEAEs that led to treatment discontinuation in more than 1 patient were periorbital edema (*n* = 3), pruritus (*n* = 3), and rash (*n* = 2). The most common treatment-related TEAEs in MOTION, such as edema and rash, were managed following individual institutional protocols. These results support the finding that TEAEs with vimseltinib are clinically manageable in the experience of the investigators, with dose interruption and reduction as the primary mitigation strategies.

This study had some limitations inherent to the study design. Due to the crossover design of MOTION, no formal comparison between vimseltinib and placebo could be performed past week 25, and COAs were completed on an open-label basis. Additionally, patients who crossed over from placebo to vimseltinib had shorter treatment duration and fewer data points while on treatment for all outcomes compared with the group randomized to vimseltinib. Furthermore, the change in the schedule of assessments during the extension period did not allow for the comparable assessment of stiffness and pain past week 49. Finally, this study was not designed to assess the ability for patients to become candidates for surgery after treatment with vimseltinib.

## Conclusion

The final results from the MOTION phase 3 trial demonstrate that vimseltinib provided robust and long-term tumor response, maintained improvements in COAs, and had a manageable safety profile with at least 2 years of follow-up on study. These long-term results further support the approval of vimseltinib in the US and EU, and they demonstrate the continued, proven functional health and symptomatic benefit provided by vimseltinib in patients with symptomatic TGCT not amenable to surgery.

## Supplementary Material

oyag421_Supplementary_Data

## Data Availability

Qualified scientific and medical researchers can address requests for de-identified individual participant data that underlie the results reported in this article at info@deciphera.com. Proposals for data will be evaluated and approved by Deciphera Pharmaceuticals, LLC, at its sole discretion. All approved researchers must sign a data access agreement before accessing the data. Data will be available as soon as possible but no later than within 1 year of the acceptance of the article for publication and for 3 years after article publication. Deciphera Pharmaceuticals, LLC, will not share data from identified participants or a data dictionary.
