# Supplementary material for: Long-term efficacy, clinical outcomes, and safety of vimseltinib in patients with tenosynovial giant cell tumor: results from the MOTION phase 3 trial
Source: Oncologist. 2026 Oct 19;31(11):oyag421. doi: 10.1093/oncolo/oyag421 (PMC13719225; doi:10.1093/oncolo/oyag421)
Supplement: Supplementary file 1 [file oyag421_supplementary_data.zip › MOTION 2-yr data cut ms_Supplementary Table 1_24June2026.docx]

**Supplementary Table 1.** Summary of TEAEs

|  | **Randomized to vimseltinib**  **n = 83** | **Crossed over to vimseltinib**  **n = 35** | **Vimseltinib total**  **N = 118** |
| --- | --- | --- | --- |
| **Any TEAE** | 83 (100) | 34 (97) | 117 (99) |
| Any treatment-related TEAE | 80 (96) | 34 (97) | 114 (97) |
| **Any grade 3/4 TEAE** | 44 (53) | 16 (46) | 60 (51) |
| Any treatment-related grade 3/4 TEAE | 31 (37) | 13 (37) | 44 (37) |
| **Any serious TEAE** | 18 (22) | 5 (14) | 23 (19) |
| Any treatment-related serious TEAE | 2 (2) | 1 (3) | 3 (3) |
| **Any TEAE leading to dose modification** | 65 (78) | 24 (69) | 89 (75) |
| Dose interruption | 55 (66) | 19 (54) | 74 (63) |
| Dose reduction | 51 (61) | 17 (49) | 68 (58) |
| **Any TEAE leading to treatment discontinuation** | 11 (13) | 3 (9) | 14 (12) |
| Any treatment-related TEAE leading to treatment discontinuation | 8 (10) | 2 (6) | 10 (8) |
| **Any TEAE leading to death** | 1 (1)^a^ | 0 | 1 (1) |

Data cutoff: February 22, 2025. Data from the safety population, which includes patients who received at least 1 dose of vimseltinib. Severity was assessed using the National Cancer Institute Common Terminology Criteria for Adverse Events, version 5.0.

**^a^**Reported reason due to “fall.”

TEAE, treatment-emergent adverse event.
