# Supplementary material for: Long-term efficacy, clinical outcomes, and safety of vimseltinib in patients with tenosynovial giant cell tumor: results from the MOTION phase 3 trial
Source: Oncologist. 2026 Oct 19;31(11):oyag421. doi: 10.1093/oncolo/oyag421 (PMC13719225; doi:10.1093/oncolo/oyag421)
Supplement: Supplementary file 1 [file oyag421_supplementary_data.zip › MOTION 2-yr data cut ms_Supplementary Figure 1_24June2026.pdf]

Supplementary Figure 1. Proportion of responses to select PROMIS items over time

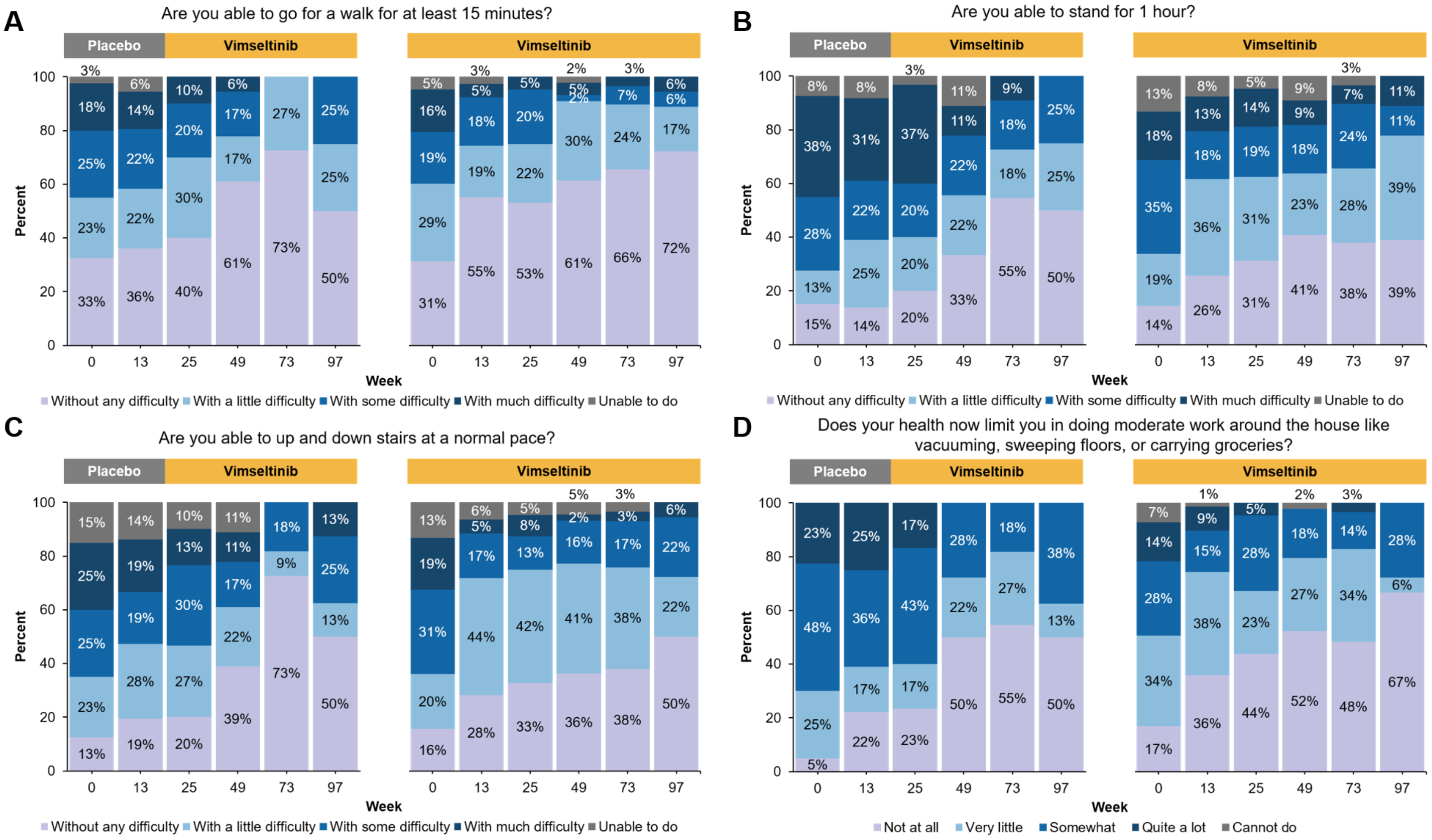

Data from the ITT population. Percentages may not total 100 due to rounding.

ITT, intent-to-treat; PROMIS, Patient-Reported Outcomes Measurement Information System.
